# Human Variation‐Informed Prioritization of MPHOSPH6 in Lung Adenocarcinoma: A Source‐Aware Multiomics Evidence Framework

**DOI:** 10.1155/humu/1879922

**Published:** 2026-09-02

**Authors:** Chongwen Fang, Min Wu, Jing Lv, Yanping Zhang, Qinyun Zheng, Xiang Shen, Shangke Huang

**Affiliations:** ^1^ Department of Gastroenterology, Anqing Municipal Hospital, Anqing, China, aqslyy.com.cn; ^2^ Department of Thoracic Surgery, West China Hospital of Sichuan University-Ziyang Hospital, Ziyang City Central Hospital, Ziyang, China; ^3^ Department of Orthopedics, West China Hospital of Sichuan University-Ziyang Hospital, Ziyang City Central Hospital, Ziyang, China; ^4^ Department of Health Management Center, Chongqing General Hospital, Chongqing University, Chongqing, China, cqu.edu.cn; ^5^ Department of Respiratory and Critical Care Medicine, The Affiliated Hospital of Southwest Medical University, Luzhou, China, ahswmu.cn; ^6^ Department of Oncology, The Affiliated Hospital of Southwest Medical University, Luzhou, China, ahswmu.cn; ^7^ Luzhou Key Laboratory of Molecular Cancer, Luzhou, Sichuan, China

**Keywords:** GWAS, human genetic variation, lung adenocarcinoma, missense variation, MPHOSPH6, multiomics biomarker, predicted gene expression, single-cell pseudobulk

## Abstract

Moving from an association signal to a clinically credible biomarker requires several links that are often conflated: verified variant identity, aligned allelic effects, reproducible gene‐level association, relevant cellular expression, and a plausible functional consequence. We developed a source‐aware multiomics framework to assess MPHOSPH6 in lung adenocarcinoma (LUAD) while keeping those evidence classes separate. Six prespecified rsIDs were recovered from the harmonized TRICL LUAD dataset, of which five reached *p* < 5 × 10 − 8. Only rs112333466 and rs76474922 were available with alignable alleles in FinnGen R10, and both showed concordant directions. Fixed‐effect estimates were OR = 1.592 for rs112333466‐T (95% CI, 1.401–1.809; *p* = 9.91 × 10 − 13) and OR = 0.819 for rs76474922‐C (95% CI, 0.773–0.867; *p* = 1.03 × 10 − 11). In a prespecified two‐variant GTEx v8 lung model, genetically predicted MPHOSPH6 expression was positively associated with LUAD in TRICL (*Z* = 3.341, *p* = 8.35 × 10 − 4) and FinnGen (*Z* = 2.697, *p* = 0.0070). This gene‐level result did not establish colocalization or connect MPHOSPH6 to the six susceptibility rsIDs. Patient‐level analysis of 89,241 immune cells from six paired tumor and normal‐adjacent lung samples found no significant difference in MPHOSPH6 pseudobulk abundance (exact paired Wilcoxon *p* = 0.3125). None of 688 lung‐lineage pharmacogenomic tests remained significant after false‐discovery‐rate correction. Ten recorded MPHOSPH6 missense alleles, including five ClinVar variants of uncertain significance, were curated; structural analysis identified I58 at an experimental RNA‐exosome interface and defined a focused perturbation series. MPHOSPH6 is therefore supported as a human–variation‐informed candidate for functional evaluation, not as a validated LUAD biomarker, pathogenic gene, drug‐response predictor, or therapeutic target.

## 1. Introduction

Lung cancer causes more cancer deaths worldwide than any other malignancy, and lung adenocarcinoma (LUAD) accounts for the largest histological fraction [1]. Genomic studies have transformed its molecular classification, but inherited susceptibility, somatic tumor alterations, and changes in the tumor microenvironment describe different biological processes [2]. Their distinction is particularly important in biomarker research. A germline association can identify a reproducible disease‐risk locus without revealing the responsible allele, the effector transcript, the relevant cell type, or the downstream phenotype. Treating each of these as an automatic consequence of the preceding observation creates a persuasive narrative but an unreliable biomarker.

Large association studies have established susceptibility regions for lung cancer and its histological subtypes, including signals near TERT‐CLPTM1L, MTAP, and the CHRNA3‐CHRNA5 locus [3]. Independent population resources such as FinnGen can test whether an allele is measurable under a different ascertainment scheme and whether its direction is consistent [4]. This comparison is informative only when the data source, genome build, coordinate, effect allele, and availability denominator are explicit. An allele that is absent from a second summary‐statistics file is not equivalent to one that is present with a discordant or null estimate.

For predominantly noncoding susceptibility signals, genetically regulated expression offers a route from sequence variation to candidate‐gene prioritization. GTEx provides tissue‐specific expression quantitative trait information for human organs, including lung [5]. S‐PrediXcan combines prediction weights and their covariance with GWAS summary statistics to test whether genetically predicted expression is associated with a phenotype [6]. The related PrediXcan framework was developed as a gene‐level association approach rather than a substitute for fine‐mapping, colocalization, or perturbational validation [7]. Sparse models, incomplete model‐variant coverage, and linkage‐disequilibrium mismatch can all affect interpretation. A directionally concordant result across cohorts strengthens prioritization, but it does not by itself establish which disease‐associated allele regulates the gene.

Orthogonal molecular data can refine a candidate, yet they introduce their own units of inference and sources of confounding. GSE139555 was designed to investigate T‐cell clonality across tumor types, not to serve as a dedicated LUAD B‐cell atlas [8]. Thousands of cells from a few individuals do not create thousands of independent patient replicates. Similarly, gene‐expression and drug‐response correlations across cancer cell lines can reflect lineage composition, growth state, or broad transcriptional programs. The GDSC resource is valuable for pharmacogenomic hypothesis generation [9], but correlation does not demonstrate target engagement or predict a response to gene perturbation. Null findings after patient‐level or lineage‐restricted analysis are therefore part of the evidence architecture, not inconvenient results to be discarded.

MPHOSPH6 encodes a component associated with the nuclear RNA exosome and has been proposed as a LUAD‐relevant candidate. We asked what level of support remains when its evidence is rebuilt from traceable human‐variation sources and analyzed at the appropriate biological scale. The study proceeded through five linked but noninterchangeable questions: whether six prespecified LUAD rsIDs could be verified and evaluated across cohorts; whether a prespecified GTEx lung model associated predicted MPHOSPH6 expression with LUAD; whether paired patient‐level single‐cell data supported a tumor‐associated expression difference; whether pan‐cancer pharmacogenomic associations persisted within lung‐lineage models; and whether recorded MPHOSPH6 missense alleles suggested experimentally testable structural sites. Susceptibility variants, prediction‐model variants, and coding alleles were deliberately maintained as separate streams. This design allowed us to identify a defensible point of gene prioritization while making the missing causal and translational links visible.

## 2. Materials and Methods

### 2.1. Study Design and Evidence Definitions

We used a source‐aware variant‐to‐biomarker design in which each conclusion was assigned to one of six evidence levels: source verification, cross‐cohort allelic support, gene‐level genetic association, patient‐level expression context, lineage‐restricted pharmacogenomic context, or computational coding‐variant prioritization. Evidence levels were not treated as interchangeable. In particular, a source‐verified variant was not described as causal; an unavailable estimate was not described as a failed replication; a predicted‐expression association was not interpreted as mediation; and a computationally prioritized substitution was not assigned pathogenicity or clinical actionability.

**Figure 1:**
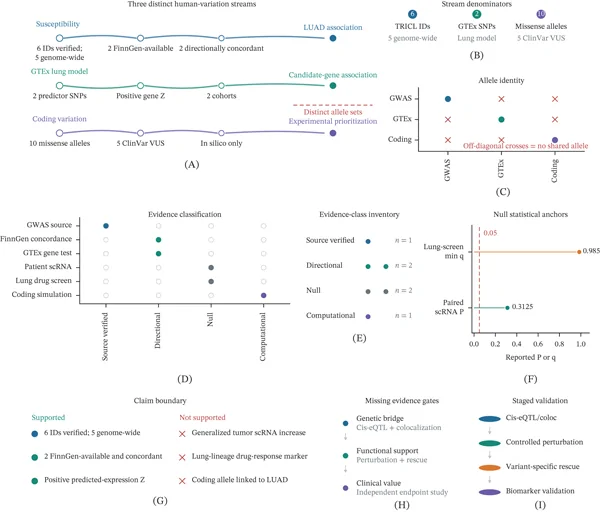
Evidence architecture and claim boundaries for human variation‐informed MPHOSPH6 prioritization. (A) Three distinct streams: prespecified LUAD susceptibility variants, GTEx lung cis‐prediction‐model variants, and recorded MPHOSPH6 coding alleles. The endpoint labels distinguish source‐level LUAD association, candidate‐gene association, and experimental prioritization. (B) Separate denominators for the six TRICL identifiers, two GTEx model variants, and ten recorded missense alleles. The values describe different source sets and are not intended for cross‐stream magnitude comparison. (C) Allele‐identity matrix; off‐diagonal crosses indicate that no allele is shared among the three streams. (D) Classification of six analytical layers as source verified, directionally supported, null, or computational. (E) Dot inventory of the number of analytical layers in each evidence class; the counts do not represent evidence strength. (F) Exact statistical anchors for the null patient‐paired single‐cell analysis and lung‐lineage pharmacogenomic screen. The dashed line denotes 0.05 and is shown only as a reference threshold. (G) Supported and unsupported claim ledger. (H) Missing genetic, functional, and clinical evidence gates. (I) Prospective validation order from cis‐eQTL/colocalization through controlled perturbation and variant‐specific rescue to independent biomarker validation. The figure synthesizes audited outputs from Figures 2, 3, 4, 5, 6, 7, and 8 and introduces no additional measured endpoint.

The complete analysis was reconstructed from publicly available, deidentified resources retrieved and audited in July 2026. Source files were processed before visualization, and each quantitative panel was generated from a persisted comma‐ or tab‐delimited output. The three human‐variation streams used in the study were (i) six prespecified LUAD susceptibility rsIDs, (ii) cis variants in the GTEx lung MPHOSPH6 expression‐prediction model, and (iii) recorded MPHOSPH6 coding alleles. Allele identity was checked across streams rather than assumed. Figure 1 summarizes the evidence architecture and the dependencies required for stronger claims.

### 2.2. LUAD GWAS Sources, Coordinate Systems, and Scan‐Level Audit

The TRICL discovery data were obtained from the GWAS Catalog harmonized summary statistics for study GCST004744 [3]. The file represents LUAD association results for 11,273 cases and 55,483 controls and provides harmonized GRCh38 coordinates. The independent dataset was FinnGen release 10 endpoint C3_NSCLC_ADENO_EXALLC, comprising 1590 cases and 314,193 controls [4]. The OpenGWAS entry ieu‐a‐984 is a GRCh37 representation of the TRICL study; it was not considered a UK Biobank dataset and was not used to align GRCh38 coordinates.

Both full autosomal summary‐statistics files were read in chunks. We recorded the total number of rows and the number satisfying *p* < 5 × 10 − 8. For genome‐wide displays, every 100th autosomal row was retained as a systematic background sample, together with all rows having *p* < 1 × 10 − 5. Candidate variants were added directly from the complete‐file lookup. A descriptive genomic‐control factor was calculated as the median one‐degree‐of‐freedom chi‐square statistic in the systematic sample divided by 0.4549364. Because systematic sampling cannot reproduce complete ancestry, imputation, relatedness, or LD‐score quality control, this factor was used only as a source‐level diagnostic.

### 2.3. Candidate‐Variant Lookup, Annotation, and Cross‐Cohort Synthesis

The following rsIDs were specified before cross‐cohort comparison: rs112333466, rs13156167, rs11133729, rs7033148, rs76474922, and rs62217816. Each identifier was searched against the complete TRICL and FinnGen files. We retained GRCh38 position, effect and other alleles, beta, standard error, *p* value, and effect‐allele frequency where available. Variant consequences were assigned with the Ensembl Variant Effect Predictor [10]. CADD PHRED values supplied with the study materials were joined after identity verification and used as annotations only [11]. The available inputs did not support reproducible fine‐mapping or posterior inclusion probabilities; no credible set or causal‐variant designation was therefore assigned.

For variants represented in both cohorts, strand and allele orientation were checked and the effects were aligned to the same effect allele. Fixed‐effect inverse‐variance meta‐analysis used wi = 1/SEi^2^ and pooled beta = *Σ*(wi betai)/*Σ*wi. Summary beta estimates were exponentiated to obtain odds ratios (ORs); 95% confidence intervals (CIs) were calculated on the log‐odds scale. Cochran Q, its chi‐square *p* value, and *I* ^2^ were reported as descriptive heterogeneity measures. With only two contributing cohorts, heterogeneity estimates were not considered stable evidence of effect modification. Genome‐wide significance was defined as *p* < 5 × 10 − 8, and false‐discovery rates in exploratory screens were controlled with the Benjamini–Hochberg procedure [12].

### 2.4. Prespecified GTEx Lung S‐PrediXcan Analysis

The GTEx v8 lung MASHR prediction database and its covariance file were obtained from PredictDB. The MPHOSPH6 model, indexed by ENSG00000135698.9, contained two GRCh38 variants. rs2303261 (chr16:82170163G>T) had a weight of −0.2015665, whereas rs72835399 (chr16:82170663G>A) had a weight of 0.0086013. Their covariance was 0.0071918, and their diagonal variances were 0.3698566 and 0.0180534, respectively.

Model variants were matched to each GWAS by chromosome and GRCh38 position, followed by effect‐allele alignment. The S‐PrediXcan statistic was calculated from the signed prediction weights, allele‐aligned GWAS Z statistics, and the GTEx covariance matrix according to the summary–predicted‐expression method [6]. We also calculated diagonal‐variance coverage, defined as the proportion of the sum of model diagonal variance represented by available variants. Both model variants were present in FinnGen. TRICL contained rs2303261 but not rs72835399; the retained variant accounted for 99.991% of diagonal model variance. The test was restricted a priori to MPHOSPH6 and was interpreted as candidate‐gene analysis rather than transcriptome‐wide discovery. Cross‐validated prediction *R* ^2^ and performance *p* values were not available for this model.

### 2.5. Patient‐Level Single‐Cell Analysis of GSE139555

The GEO record and deposited files for GSE139555 were audited before sample selection [13]. The full dataset contained 200,626 cells from 14 patients and 32 tumor, normal‐adjacent tissue (NAT), or blood samples. It was generated with single‐cell RNA sequencing and T‐cell receptor sequencing to study clonal T‐cell expansion across several cancer types [8]. The lung subset comprised six patients, each contributing paired tumor and NAT samples. We used all 12 lung 10x gene‐by‐cell count matrices and the deposited integrated metadata.

Raw MPHOSPH6 counts and total unique molecular identifier counts were recovered for each lung cell and matched to deposited cell identifiers. For visualization, expression was represented as log1p(counts per 10,000), and the deposited UMAP coordinates were used without reinterpreting the original clustering. Inference was performed at patient level. For each sample, pseudobulk abundance was calculated as 10^6^ × *Σ*(MPHOSPH6 counts)/*Σ*(all − gene counts); detection fraction was the proportion of cells with at least one MPHOSPH6 count. Paired tumor and NAT values were compared using an exact two‐sided paired Wilcoxon signed‐rank test. A paired t test served as a sensitivity analysis. No cell‐level *p* value was used to support a tissue difference, and no B‐cell–specific analysis was inferred from this pan‐immune subset.

### 2.6. Expression Atlas‐GDSC Pharmacogenomic Analysis

Baseline MPHOSPH6 transcript‐per‐million (TPM) values were obtained from Expression Atlas study E‐MTAB‐3983 [14]. Drug‐response records were obtained from the GDSC1 and GDSC2 fitted dose‐response files, Release 8.5 dated 27 October 2023 [9]. Cell‐line identifiers were normalized by converting names to uppercase and removing nonalphanumeric characters. The pharmacological endpoint was natural‐log half‐maximal inhibitory concentration (LN_IC50). This analysis therefore linked transcript abundance to an observational response metric; it did not measure MPHOSPH6 protein, genetic dependency, or direct drug‐target engagement.

Spearman correlations were calculated separately for every GDSC dataset‐drug combination. Pan‐cancer tests required at least 50 matched cell lines. To assess lineage dependence, the analysis was repeated among lung‐lineage cell lines, requiring at least 15 matched lines per test. The 28 records corresponding to a prespecified compound subset were also retained for direct audit. *p* values were adjusted with the Benjamini–Hochberg procedure within the complete pan‐cancer and lung‐lineage screens [12]. A positive rho indicates that greater MPHOSPH6 TPM accompanied a higher LN_IC50; this direction was not labeled resistance, and a negative rho was not labeled sensitivity because neither implies a perturbational MPHOSPH6 effect.

### 2.7. Human Coding‐Variant Curation and In Silico Prioritization

MPHOSPH6 coding coordinates were defined with NM_005792.2/NP_005783.2 and UniProt Q99547, which encode a 160‐amino‐acid protein. Missense records were retrieved from NCBI dbSNP [15], and clinical assertions were checked against ClinVar [16]. At multiallelic sites, frequencies were retained for the specific alternate allele and source population. A dbSNP identifier was treated as evidence of cataloging, whereas a ClinVar variant of uncertain significance (VUS) was retained as an uncertain classification rather than a pathogenic label.

Sequence‐context estimates were obtained with ESM‐2 esm2_t12_35M_UR50D for all 19‐nonreference amino acids at each of 160 positions, yielding 3040 substitutions [17]. The reported score was the alternate‐residue log likelihood minus the reference‐residue log likelihood at the same position. These scores provide a model‐dependent ranking and are not calibrated probabilities of pathogenicity. Structural confidence and full‐length residue context were taken from the AlphaFold Protein Structure Database model for Q99547 [18].

Experimentally resolved interfaces were mapped in PDB 6D6Q, the cryo‐electron microscopy structure of the human nuclear RNA exosome‐MTR4 complex containing MPHOSPH6 [19]. A partner residue was considered in contact when any heavy atom was within 5 Å of an MPHOSPH6 heavy atom. EvoEF2 was used to estimate changes in total energy for the recorded missense alleles and changes in interface binding energy for all 19 substitutions at I58 [20]. Values are reported in EvoEF2 internal energy units and were not interpreted as measured free‐energy changes. The known participation of MPHOSPH6 in nuclear RNA‐exosome biology informed the choice of later functional readouts [21] but was not used to assign a cellular phenotype to any variant.

### 2.8. Statistical Analysis and Reproducibility Controls

All tests were two‐sided. GWAS beta coefficients were handled on the log‐odds scale. Exact paired Wilcoxon tests, paired *t* tests, Spearman correlations, fixed‐effect meta‐analysis, heterogeneity statistics, and Benjamini–Hochberg adjustment were implemented in Python 3.12 with NumPy, pandas, and SciPy. Figures were generated with Matplotlib from persisted analysis tables rather than from manually entered values.

Reproducibility checks were defined before final figure assembly. They required recovery of all six prespecified rsIDs in TRICL, exactly two in FinnGen, concordant directions for those two alleles, positive MPHOSPH6 S‐PrediXcan Z statistics in both cohorts, nonsignificant patient‐level paired single‐cell tests, no lung‐lineage GDSC association with *q* < 0.05, and five ClinVar VUS among the 10 curated missense alleles. Figure‐level assertions additionally checked sample sizes, panel counts, labels, statistical anchors, source‐table fields, and consistency between vector and raster exports.

## 3. Results

### 3.1. The Two LUAD GWAS Resources Differed Substantially in Scale and Scan Characteristics

**Figure 2:**
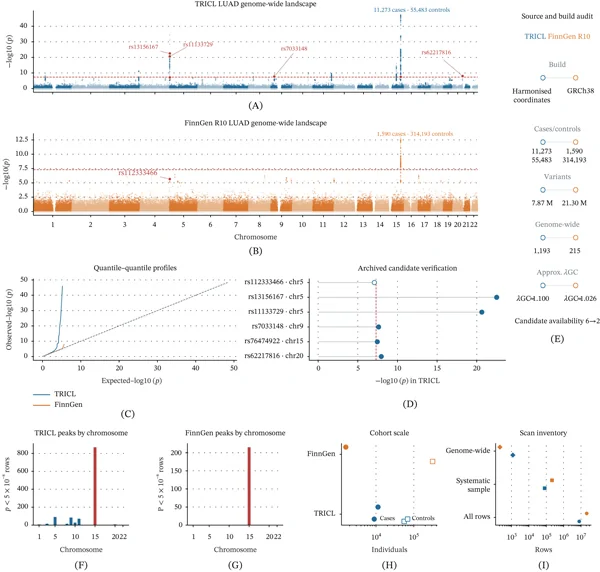
Provenance and scan‐level audit of the two LUAD GWAS resources. (A, B) Genome‐wide association landscapes for harmonized GCST004744/TRICL and FinnGen R10. Systematically sampled background variants are displayed with all retained genome‐wide significant rows; highlighted points denote prespecified candidate rsIDs when present. The dashed line marks *p* = 5 × 10 − 8. (C) Quantile–quantile profiles from the systematic autosomal samples. (D) TRICL *p* values for the six prespecified candidate identifiers; the open point denotes rs112333466, which did not reach the genome‐wide threshold. (E) Source, coordinate build, sample size, full row count, genome‐wide row count, and approximate genomic‐control audit. (F, G) Chromosomal distribution of genome‐wide significant rows in TRICL and FinnGen. Counts represent summary‐statistics rows, not independent loci. (H) Case and control sample sizes. (I) Full‐scan, systematic‐sample, and genome‐wide‐significant row inventories. Approximate lambda values are descriptive sample‐based diagnostics and do not replace full cohort quality control.

The harmonized TRICL file contained 7,870,055 rows, of which 1193 met *p* < 5 × 10 − 8. The FinnGen R10 file contained 21,303,860 rows and 215 genome‐wide significant rows (Figure 2A, B, F, G, and I). These counts refer to summary‐statistics rows rather than independent susceptibility loci. The difference was accompanied by distinct sample structures: TRICL included 11,273 cases and 55,483 controls, whereas FinnGen included 1590 cases and 314,193 controls (Figure 2E,H).

Quantile‐quantile profiles from the systematic autosomal samples yielded approximate genomic‐control factors of 1.100 for TRICL and 1.026 for FinnGen (Figure 2C,E). Because these estimates were based on a sampling audit, they were not used to claim the absence of population structure or other cohort‐specific bias. Instead, the two resources were kept separate throughout genome‐wide visualization and candidate lookup. The coordinate audit further confirmed that the GWAS Catalog‐harmonized TRICL data and the native FinnGen data were both evaluated in GRCh38, avoiding direct use of GRCh37 coordinates from the OpenGWAS representation.

### 3.2. Cross‐Cohort Evidence Was Available for Two of the Six Prespecified Susceptibility Variants

**Figure 3:**
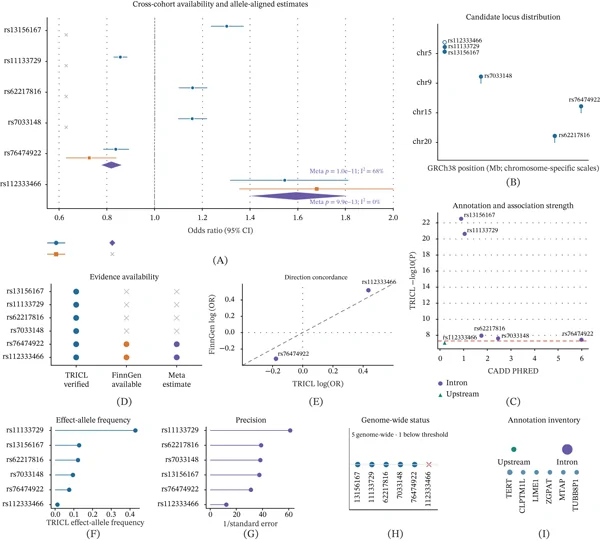
Identity verification and cross‐cohort evaluation of six prespecified susceptibility variants. (A) Allele‐aligned ORs and 95% CIs for TRICL and, when available, FinnGen; fixed‐effect diamonds are displayed only for the two alleles represented in both cohorts. Gray crosses mark unavailable FinnGen estimates and are not null effects. (B) GRCh38 positions shown on chromosome‐specific scales. (C) TRICL association strength in relation to the supplied CADD PHRED annotation, with VEP consequence class encoded by shape. (D) Cohort availability matrix. (E) Comparison of TRICL and FinnGen log‐odds effects for the two‐shared variants. (F) TRICL effect‐allele frequencies. (G) Inverse‐standard‐error context. (H) Genome‐wide‐threshold classification in TRICL; five variants satisfy *p* < 5 × 10 − 8 and rs112333466 does not. (I) VEP consequence and overlapping‐gene inventory. CADD values are annotations and do not establish pathogenicity.

Complete‐file lookup recovered all six prespecified rsIDs from TRICL (Figure 2D and Figure 3A, D, and H). Five crossed the conventional genome‐wide threshold. The effect estimates were rs13156167‐C near TERT, OR = 1.303 and *p* = 3.10 × 10 − 23; rs11133729‐G in CLPTM1L, OR = 0.856 and *p* = 2.30 × 10 − 21; rs7033148‐G near MTAP, OR = 1.157 and *p* = 2.37 × 10 − 8; rs76474922‐C in the CHRNA3‐CHRNA5 region, OR = 0.838 and *p* = 3.47 × 10 − 8; and rs62217816‐T near LIME1‐ZGPAT, OR = 1.158 and *p* = 1.09 × 10 − 8. rs112333466‐T near TERT was detectable and showed OR = 1.545, but its *p* value of 8.11 × 10 − 8 did not satisfy *p* < 5 × 10 − 8.

Only rs112333466 and rs76474922 were represented in FinnGen at matching GRCh38 coordinates with alleles that could be aligned (Figure 3A,D). Their directions agreed with TRICL. For rs112333466‐T, the FinnGen estimate was OR = 1.681 (*p* = 2.14 × 10 − 6); for rs76474922‐C, it was OR = 0.727 (*p* = 1.31 × 10 − 5). The corresponding fixed‐effect estimates were OR = 1.592 for rs112333466‐T (95% CI, 1.401–1.809; *p* = 9.91 × 10 − 13; *I* ^2^ = 0*%*) and OR = 0.819 for rs76474922‐C (95% CI, 0.773–0.867; *p* = 1.03 × 10 − 11; *I* ^2^ = 68.3*%*) (Figure 3A,E). Cochran′s Q test for rs76474922 gave *p* = 0.0759. With two cohorts, the apparent heterogeneity was treated as a caution rather than a stable quantitative estimate.

VEP classified five of the six candidates as intronic and one as upstream (Figure 3C,I). Their genomic positions, effect‐allele frequencies, association strength, and estimate precision are summarized in Figure 3B, F–H). Supplied CADD PHRED scores were modest and did not alter the statistical evidence class. Thus, six rsIDs were verified in TRICL, but the denominator for cross‐cohort comparison was two rather than six. The other four were unavailable in FinnGen and could not be counted as either replicated or nonreplicated.

### 3.3. Predicted Lung MPHOSPH6 Expression Showed the Same Association Direction in TRICL and FinnGen

**Figure 4:**
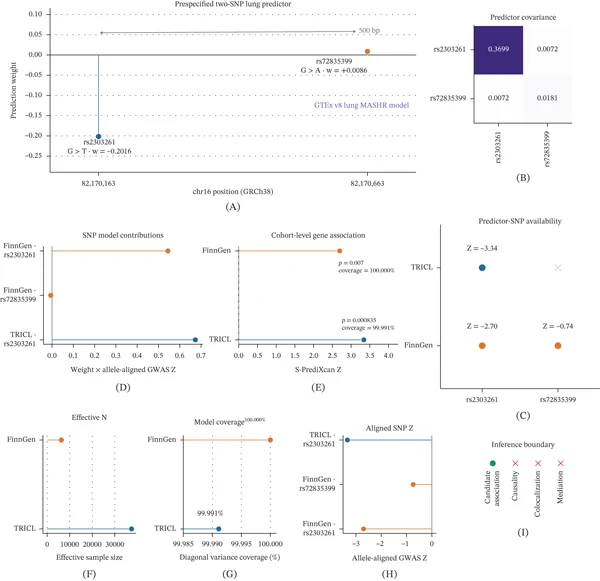
Prespecified GTEx v8 lung model of genetically predicted MPHOSPH6 expression. (A) GRCh38 coordinates and signed weights of the two MASHR predictor variants. (B) Predictor covariance matrix. (C) Variant availability and allele‐aligned GWAS Z statistics by cohort. (D) Per‐variant weight‐by‐Z contributions. (E) Cohort‐level S‐PrediXcan Z statistics, *p* values, and diagonal‐variance coverage. (F) Effective sample sizes. (G) Predictor variance coverage. (H) Allele‐aligned Z statistics for the model variants. (I) Inference boundary. The analysis supports a prespecified gene‐level association; it does not establish a causal allele, colocalization, or mediation of the six susceptibility candidates.

The GTEx v8 lung MPHOSPH6 model contained rs2303261 and rs72835399, separated by 500 bp on chromosome 16, with strongly asymmetric prediction weights (Figure 4A,B). FinnGen supplied allele‐aligned estimates for both. TRICL supplied rs2303261 but not the lower‐variance rs72835399 term (Figure 4C). The negative weight for rs2303261 combined with a negative aligned GWAS Z statistic in each cohort, creating a positive contribution to the predicted‐expression association (Figure 4D,H).

The resulting gene‐level statistic was positive in both datasets: *Z* = 3.341 (*p* = 8.35 × 10 − 4) in TRICL and *Z* = 2.697 (*p* = 0.0070) in FinnGen (Figure 4E). TRICL retained 99.991% of the model′s diagonal variance despite the missing second predictor, whereas FinnGen coverage was complete (Figure 4F,G). Directional agreement therefore supported MPHOSPH6 for follow‐up under this prespecified lung model.

This association was not an allele‐level bridge to the six susceptibility candidates. Those rsIDs are located on chromosomes 5, 9, 15, and 20, whereas the prediction model uses two cis variants on chromosome 16. No candidate allele was shared between the two streams. Without a complete cis‐eQTL analysis, colocalization, and conditional evaluation, the gene‐level signal cannot be interpreted as mediation of the verified susceptibility associations (Figure 4I).

### 3.4. Paired Patient‐Level Analysis Did Not Support a Consistent Tumor‐Associated Expression Shift

**Figure 5:**
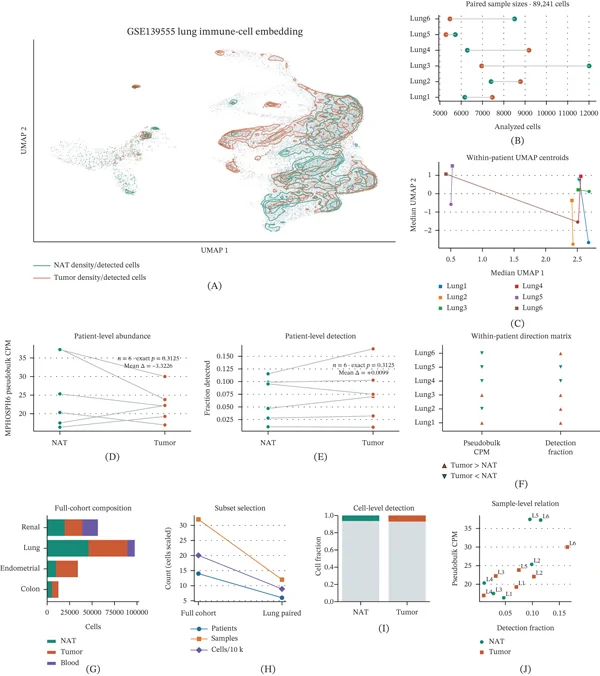
Patient‐level analysis of MPHOSPH6 in the paired GSE139555 lung subset. (A) UMAP embedding of 89,241 lung immune cells with tissue‐specific density contours and MPHOSPH6‐detected cells overlaid for descriptive localization. (B) Paired tumor and NAT cell counts for six patients. (C) Tumor and NAT UMAP centroids within each patient. (D, E) Paired pseudobulk CPM and detection fraction; exact two‐sided paired Wilcoxon *p* values are displayed. (F) Direction of the within‐patient difference for each endpoint; the two measurement units are kept separate. (G) Composition of the complete deposited cohort by organ and sample source. (H) Selection from the full cohort to the paired lung subset. (I) Detected and zero‐count cell composition. (J) Sample‐level relationship between pseudobulk CPM and detection fraction. The inferential unit is the patient, not the cell.

The deposited GSE139555 cohort comprised 200,626 cells from 14 patients and 32 samples distributed across organs and source tissues (Figure 5G,H). Restriction to paired lung tissues yielded 89,241 immune cells from six patients, with one tumor and one NAT sample per patient (Figure 5A,B). UMAP density contours overlapped broadly between tumor and NAT, and the paired centroid shifts varied among individuals (Figure 5A,C). These plots were used to describe the data structure rather than to provide cell‐level inference.

At the patient level, mean MPHOSPH6 pseudobulk abundance was 25.71 counts per million (CPM) in NAT and 22.39 CPM in tumor. The mean paired tumor‐minus‐NAT difference was −3.32 CPM. Neither the exact paired Wilcoxon test (*p* = 0.3125) nor the paired *t* test (*p* = 0.2795) supported a systematic difference (Figure 5D). Detection fractions averaged 0.0659 in NAT and 0.0758 in tumor, with a mean paired difference of 0.00995; the exact Wilcoxon *p* value was 0.3125 and the paired *t*‐test *p* value was 0.3535 (Figure 5E).

Individual patients did not share a uniform direction for either endpoint (Figure 5F). The sample‐level relation between pseudobulk CPM and detection fraction, together with the large zero‐count fraction, further showed why the two summaries should be considered jointly (Figure 5I,J). On this evidence, GSE139555 does not support a generalized increase of MPHOSPH6 in tumor immune cells. It also provides no basis for a B–cell‐specific mechanism.

### 3.5. Apparent Pan‐Cancer Pharmacogenomic Associations Disappeared After Lung‐Lineage Restriction

**Figure 6:**
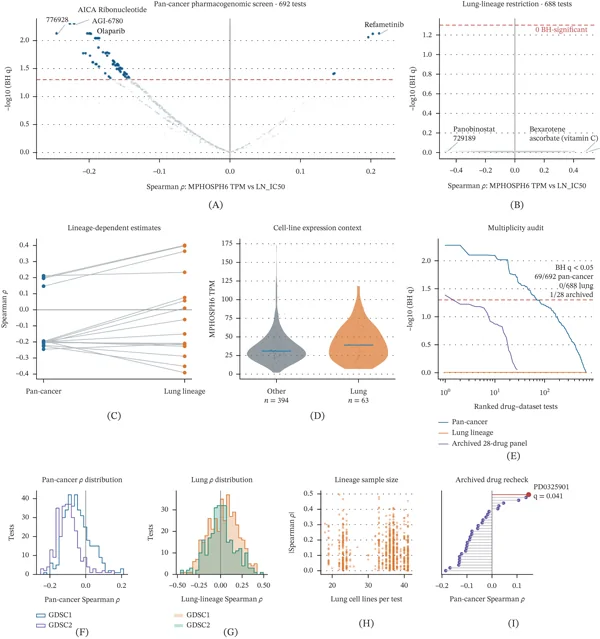
Lineage sensitivity of Expression Atlas‐GDSC pharmacogenomic associations. (A) Pan‐cancer Spearman associations between MPHOSPH6 TPM and GDSC LN_IC50 across 692 drug‐dataset tests. (B) Corresponding results for 688 tests restricted to lung‐lineage cell lines; none remains significant after Benjamini–Hochberg correction. Positive and negative rho values indicate association direction only and are not labeled resistance or sensitivity. (C) Change from pan‐cancer to lung‐lineage estimates for selected compounds. (D) Cell‐line expression and matching context. (E) Ranked adjusted–*p*‐value curves and screen counts. (F, G) Pan‐cancer and lung‐lineage rho distributions by GDSC release. (H) Lung‐lineage sample size per test in relation to absolute rho. (I) Audit of the prespecified 28 drug‐dataset records. Only the pan‐cancer GDSC2 PD0325901 record has *q* < 0.05, and it is not significant in the lung‐lineage analysis. The figure does not support a therapeutic‐target or drug‐response claim.

Expression Atlas supplied MPHOSPH6 TPM measurements for 457 cancer cell lines. Of these, 401 matched at least one GDSC drug‐response record, including 44 lines annotated as lung lineage (Figure 6D). The pan‐cancer screen comprised 692 drug‐dataset correlations. Sixty‐nine had *q* < 0.05, although the rho estimates were generally modest and extended in both positive and negative directions (Figure 6A, E, and F). This pattern is compatible with a mixture of lineage‐related and drug‐specific effects rather than a simple MPHOSPH6 response phenotype.

The lung‐lineage analysis retained 688 tests meeting the minimum sample‐size rule. None survived false‐discovery‐rate correction; the smallest *q* value was 0.985 (Figure 6B, E, G, and H). For selected compounds, both the magnitude and direction of rho changed when the analysis was restricted from all cancer cell lines to lung‐lineage models (Figure 6C). Among the prespecified 28 drug‐dataset records, the only pan‐cancer result with *q* < 0.05 was PD0325901 in GDSC2 (rho = 0.147, *q* = 0.041). It did not remain significant in the lung‐restricted analysis (Figure 6I).

These results do not establish that MPHOSPH6 abundance predicts drug sensitivity or resistance in LUAD. They instead demonstrate that the apparent pan‐cancer associations are not robust to the disease‐relevant lineage restriction applied here.

### 3.6. Recorded MPHOSPH6 Missense Alleles Formed a Separate Human‐Variation Dataset

**Figure 7:**
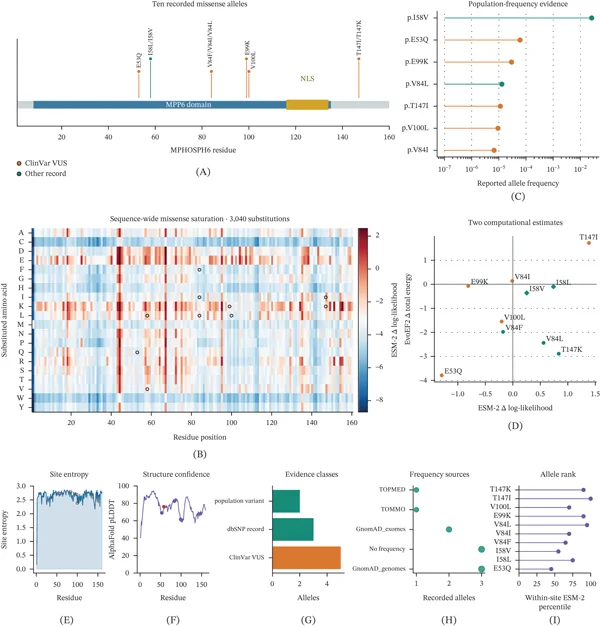
Recorded MPHOSPH6 missense variation and computational sequence context. (A) Protein‐position needle plot of 10 recorded missense alleles. (B) ESM‐2 saturation landscape for 3040 nonreference substitutions; open circles identify recorded alleles. (C) Reported alternate‐allele frequencies on a logarithmic scale; frequency resources and source populations differ among records. (D) ESM‐2 delta log likelihood versus EvoEF2 change in total energy for recorded alleles; diamonds denote alleles at an experimentally resolved interface. (E) Position‐wise ESM‐2 site entropy. (F) AlphaFold pLDDT context. (G) Evidence‐class inventory. (H) Frequency‐source inventory. (I) Within‐position ESM‐2 percentile for each recorded allele. ClinVar VUS designations indicate uncertainty, and neither computational score constitutes functional or clinical validation.

Ten missense alleles were mapped to the 160‐residue MPHOSPH6 reference sequence: p.E53Q, p.I58L, p.I58V, p.V84F, p.V84I, p.V84L, p.E99K, p.V100L, p.T147I, and p.T147K (Figure 7A). Five records carried a ClinVar VUS classification: p.E53Q, p.V84I, p.E99K, p.V100L, and p.T147I (Figure 7G). p.I58V had the highest reported allele frequency, 0.0262. Other frequency‐annotated alleles were uncommon, but the source population and reporting resource varied among records (Figure 7C,H). None of the 10 alleles was one of the six LUAD susceptibility rsIDs, and none was classified as pathogenic.

The ESM‐2 saturation analysis generated estimates for all 3040 possible single‐residue substitutions. The distribution varied by position and alternate amino acid, indicating nonuniform sequence constraint across the protein (Figure 7B,E). Recorded alleles occupied different positions within those local distributions (Figure 7I). AlphaFold confidence also varied along the sequence and provided an independent structural‐context track (Figure 7F).

ESM‐2 sequence scores and EvoEF2 total‐energy changes were not consistently aligned for the recorded alleles (Figure 7D). p.T147I, for example, had a positive ESM‐2 score of 1.384 but an unfavorable EvoEF2 total‐energy change of 1.72 units. p.E53Q showed the opposite pattern, with an ESM‐2 score of −1.292 and an EvoEF2 estimate of −3.79 units. The discordance reinforced the need to treat both models as prioritization tools. Neither provides a clinical classification, and agreement between them would still require experimental validation.

### 3.7. The Experimental RNA‐Exosome Structure Identified I58 as a Tractable Perturbation Site

**Figure 8:**
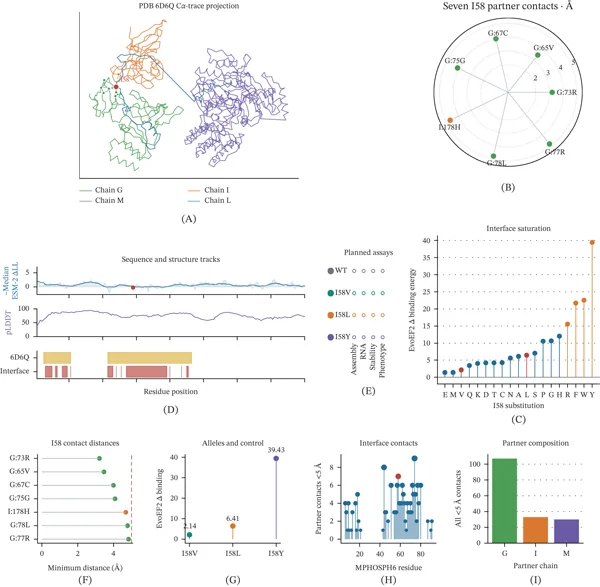
Experimental RNA‐exosome interface mapping and I58 perturbation design. (A) Two‐dimensional principal‐axis projection of PDB 6D6Q C‐alpha traces. The display is a projection, not a molecular surface or a dynamic simulation; MPHOSPH6 chain L and I58 are highlighted. (B) Radial map of seven partner residues within 5 Å of I58. (C) EvoEF2 interface‐saturation estimates for all 19 I58 substitutions. (D) Separate sequence‐constraint, AlphaFold‐confidence, experimental‐coverage, and interface‐contact tracks. (E) Prospective four‐construct by four‐endpoint assay matrix; hollow symbols indicate experiments that have not been performed. (F) Ranked heavy‐atom distances between I58 and partner residues. (G) Recorded I58V and I58L alleles and the unrecorded predicted‐disruptive I58Y control. (H) Contact landscape across the experimentally resolved MPHOSPH6 interface. (I) Partner‐chain composition. EvoEF2 reports internal computational energy units, not measured free‐energy changes.

The segment of MPHOSPH6 resolved in PDB 6D6Q occupied a multichain RNA‐exosome interface (Figure 8A, H, and I). I58 was positioned within that experimental segment and had seven partner residues within 5 Å. The shortest heavy‐atom separation was 3.21 Å (Figure 8B,F). Interface saturation with EvoEF2 yielded relatively modest estimates for several conservative replacements and larger unfavorable values for aromatic substitutions. I58Y, I58W, and I58F ranked among the most disruptive predictions (Figure 8C).

Sequence constraint, AlphaFold confidence, experimental coverage, and direct‐contact status were displayed as separate tracks rather than collapsed into one composite pathogenicity score (Figure 8D). Together they supported I58 as an experimentally accessible site, not as a disease‐causing residue. The recorded I58V and I58L alleles also provided naturally occurring comparators, whereas the unrecorded I58Y substitution supplied a predicted‐disruptive positive control (Figure 8G).

We therefore specified a four‐construct series comprising wild type, I58V, I58L, and I58Y. Exosome assembly, RNA processing, RNA stability, and proliferation were selected as prospective readouts (Figure 8E). The assays have not been performed; the hollow symbols in the figure denote planned measurements. This design is intended to test the ranking behavior of the sequence and interface models while separating a common population allele, a second recorded allele, and a deliberately disruptive control.

### 3.8. Integrated Evidence Supported Prioritization but Exposed the Missing Biomarker Links

The final evidence map began with three nonoverlapping human‐variation streams (Figure 1A). The susceptibility stream comprised six prespecified LUAD candidate identifiers verified in TRICL, five of which met the conventional genome‐wide threshold; two were represented in FinnGen and both had concordant effect directions. The GTEx lung MPHOSPH6 prediction model comprised two predictor variants and yielded a positive gene‐level statistic in both cohorts. The coding stream comprised 10 recorded missense alleles, including five ClinVar variants of uncertain significance, and remained computational. These denominators were displayed separately because they describe different source populations and cannot be compared as evidence magnitudes (Figure 1B). The allele‐identity matrix confirmed that no variant was shared among the susceptibility, prediction‐model, and coding streams (Figure 1C).

Across the six analytical layers, the GWAS‐source audit was classified as source verified; FinnGen concordance and the prespecified GTEx gene test were classified as directionally supportive; the patient‐paired single‐cell analysis and lung‐lineage drug screen were null; and the coding‐variant simulation remained computational (Figure 1D,E). The two null anchors were the exact paired single‐cell *p* value of 0.3125 and the smallest lung‐lineage pharmacogenomic *q* value of 0.985 (Figure 1F). These results do not establish equivalence or exclude smaller context‐specific effects, but they do prevent the available single‐cell and pharmacogenomic data from being used as positive validation.

The resulting claim ledger supports source verification of the six candidate identifiers, directional agreement for the two FinnGen‐available susceptibility alleles, and a positive prespecified predicted‐expression association. It does not support generalized tumor elevation, a lung‐lineage drug‐response marker, or linkage of a recorded coding allele to LUAD (Figure 1G). A genetic bridge would require complete MPHOSPH6 cis‐eQTL, conditional‐association, and colocalization analyses; functional support would require controlled perturbation and variant‐specific rescue; and biomarker evaluation would require an independently powered study with a prespecified clinical endpoint (Figure 1H). The prospective order is therefore cis‐eQTL and colocalization, controlled perturbation, variant‐specific rescue, and only then independent biomarker validation (Figure 1I). This hierarchy supports continued prioritization of MPHOSPH6 without presenting it as a causal gene, pathogenic coding locus, validated biomarker, or therapeutic target.

## 4. Discussion

This study addresses a recurrent problem in variant‐to‐biomarker research: Several valid observations can be combined into an invalid causal story when their alleles, biological scales, and inferential units are not tracked. Reanalysis of the MPHOSPH6 evidence produced a narrower but more defensible conclusion. Six prespecified LUAD rsIDs were verifiable in TRICL; two were evaluable in FinnGen with concordant directions, and a lung‐specific MPHOSPH6 predicted‐expression model gave positive gene‐level associations in both cohorts. These results justify continued investigation of MPHOSPH6. They do not establish that the verified susceptibility variants regulate MPHOSPH6 or that the gene is already a LUAD biomarker.

The candidate‐variant analysis illustrates why availability must be part of a replication statement. All six identifiers existed in TRICL, but one fell short of the conventional genome‐wide threshold. Four were absent from the FinnGen endpoint file, leaving only two eligible for direct cross‐cohort comparison. Calling the other four nonreplicated would imply that a usable estimate contradicted or failed to reproduce the discovery result; no such test was possible. The two‐shared variants did show directional agreement, and the pooled estimates were precise. Even so, the meta‐analysis contained only two cohorts, and the *I* ^2^ estimate for rs76474922 was unstable. Source verification and partial cross‐cohort support are accurate descriptions; six causal variants are not.

The predicted‐expression analysis provides a separate reason to prioritize MPHOSPH6. Its consistent direction in TRICL and FinnGen argues against an association created solely by one cohort′s ascertainment. The sparse model also carried unusually high diagonal‐variance coverage in both datasets because rs2303261 accounted for most predicted variance. Those strengths do not remove the central limitation: the two model variants are not the six susceptibility candidates. Moreover, model performance statistics were unavailable, and a single‐gene S‐PrediXcan test cannot establish colocalization or mediation. The next genetic step is therefore not to append a stronger causal adjective. It is to analyze the full MPHOSPH6 cis region with adequately matched lung and cell‐type eQTL data, perform conditional and colocalization analyses, and assess whether the gene association is independent of neighboring chromosome 16 signals.

The single‐cell reanalysis changed the interpretation by restoring the patient as the unit of inference. The lung subset contained 89,241 immune cells, but they came from six paired individuals. Cell‐level tests would overstate precision because cells from one patient share exposure, sampling, processing, and biological history. At pseudobulk level, neither abundance nor detection fraction differed significantly between tumor and NAT, and paired directions were heterogeneous. This finding does not exclude a subtle state‐specific effect in a larger or differently sampled cohort. It does show that GSE139555 cannot support generalized tumor elevation or a B‐cell‐specific MPHOSPH6 mechanism. A future single‐cell study should enroll more patients, prespecify epithelial and immune populations, use patient‐aware models, and include spatial or protein‐level replication.

The pharmacogenomic results provide a second example of context changing the conclusion. Numerous pan‐cancer correlations passed multiplicity correction, but the effect sizes were small and did not persist in the lung‐lineage screen. Restricting lineage reduces the chance that tissue‐of‐origin differences in expression and drug response generate a spurious relationship, although it also reduces power. The resulting null screen cannot prove that MPHOSPH6 has no pharmacological relevance. It does mean that current observational data are insufficient to call MPHOSPH6 a drug‐response marker. Such a claim would require direct perturbation, confirmation of altered MPHOSPH6 abundance or function, reproducible dose‐response shifts across several LUAD models, and a rescue or orthogonal validation experiment.

The coding‐variant module places the work more directly within human mutation research, but only if its independence is preserved. The 10 missense alleles are recorded human variants, five are ClinVar VUS, and none is one of the LUAD susceptibility variants. VUS denotes unresolved clinical significance, not intermediate pathogenicity. The sequence and energy models supply complementary rankings rather than a clinical verdict, as shown by their discordant estimates for several recorded alleles. I58 is notable because it lies in an experimentally observed interface, not because a simulation proved that it causes disease. Comparing I58V and I58L with wild type and a predicted‐disruptive I58Y control would test both allele‐specific function and the validity of the computational ranking. Until those assays are performed, the appropriate output is an experimental priority list.

The study′s contribution is therefore methodological as well as gene specific. A source‐aware framework makes the denominator of each claim visible: six variants were verifiable in TRICL, two were testable in FinnGen, two different variants formed the expression model, six patients contributed paired single‐cell data, and 44 lung‐lineage cell lines underpinned the restricted pharmacogenomic analysis. It also prevents evidence inflation across modalities. Predicted expression is a genetic association; single‐cell abundance is an observational cellular measurement; an expression‐LN_IC50 correlation is a context‐dependent association; and a sequence or structural score is a computational estimate. Multiomics integration becomes more credible when these meanings are retained rather than averaged into a single support score.

Several limitations remain. Both GWAS resources largely represent European‐ancestry populations, so ancestry transferability was not evaluated. The displayed genomic‐control factors were approximate scan diagnostics, not replacements for cohort‐level imputation, ancestry, relatedness, or LD‐score assessments. Four candidate variants could not be assessed in FinnGen, and the two‐cohort meta‐analysis offered limited heterogeneity resolution. The S‐PrediXcan analysis was prespecified for one gene, used a sparse model, lacked reported cross‐validated performance, and was not accompanied by colocalization. The single‐cell analysis included only six paired lung patients and evaluated overall immune‐cell abundance rather than adequately powered cell‐type‐specific effects. The GDSC lung‐lineage subset was observational and smaller than the pan‐cancer set. Finally, ESM‐2 and EvoEF2 estimates have not been calibrated against experimental MPHOSPH6 variant effects.

These limitations define a staged validation path. First, the genetic association should be examined in an independent ancestry or additional LUAD resource, followed by complete cis‐eQTL and colocalization analysis. Second, controlled MPHOSPH6 perturbation should measure RNA‐exosome assembly and RNA‐processing phenotypes in lung epithelial and relevant immune models. Third, the I58 series should be tested with matched protein‐expression controls and rescue experiments. Only after the genetic and functional gates are satisfied should a clinical study assess whether MPHOSPH6 adds reproducible value to an established LUAD endpoint. This order keeps the work translational without presenting an unvalidated candidate as a biomarker.

## 5. Conclusion

A source‐aware multiomics analysis verified six prespecified LUAD rsIDs in TRICL, identified concordant effects for the two alleles available in FinnGen, and reproduced the positive direction of a prespecified GTEx lung MPHOSPH6 predicted‐expression association in both cohorts. Patient‐level single‐cell and lung‐lineage pharmacogenomic analyses did not support generalized tumor elevation or drug‐response prediction. Human coding‐variant curation and the experimental 6D6Q interface nominate I58 for functional testing, but neither pathogenicity nor LUAD causality has been established. The evidence supports MPHOSPH6 as a human–variation‐informed candidate for staged genetic and experimental validation.

## Author Contributions

C.F., M.W., and J.L. have contributed to the work equally and should be regarded as co‐first authors. C.F. and M.W. conceived and designed the study, performed the bioinformatics analyses, interpreted the data, and drafted the manuscript. J.L. and Y.Z. contributed to data collection, statistical analysis, and figure preparation. Q.Z., X.S., and S.H. supervised the work, critically revised the manuscript, and contributed to interpretation.

## Funding

This study was supported by the Anhui Provincial Health Commission (AHWJ2023BAa20028); the Key Research Project of the Anhui Provincial Department of Education (2025AHGXZK31479); the Ziyang Science and Technology Association/Medical Association Funded Project (2025129); the Higher Education Teaching Reform and Research Program of Southwest Medical University (JG2024033); the Applied Basic Research Project of Southwest Medical University (No. 2025JC100); and the Science and Technology Strategic Cooperation Programs of Luzhou Municipal People′s Government and Southwest Medical University (2023LZXNYDJ033).

## Disclosure

All authors read and approved the final manuscript.

## Ethics Statement

All analyses used publicly available, deidentified datasets. No participants were newly recruited, and no identifiable individual‐level data were collected. Additional institutional ethics approval was therefore not required for this secondary analysis.

## Consent

The authors have nothing to report.

## Conflicts of Interest

The authors declare no conflicts of interest.

## Data Availability

The study used GWAS Catalog study GCST004744, FinnGen release 10, PredictDB GTEx v8 lung MASHR models, GEO GSE139555, Expression Atlas E‐MTAB‐3983, GDSC Release 8.5, dbSNP, ClinVar, AlphaFold DB, and PDB 6D6Q. Analysis scripts, persisted derived tables, the executed verification notebook, the panel‐level source map, and all eight main figures are included with the manuscript materials.
